# Global Trends Towards Population Health Management and Key Lessons and Initiatives in the Singapore Context

**DOI:** 10.5334/ijic.7016

**Published:** 2022-09-16

**Authors:** Maria Abraham, Ming Jing Lim, Woan Shin Tan, Jason Cheah

**Affiliations:** 1 Accountable Care Strategy, Group Accountable Care, National Healthcare Group, Singapore; 2 Population Health Management, Group Accountable Care, National Healthcare Group, Singapore; 3 Health and Services & Outcomes Research, National Healthcare Group, Singapore; 4 Group Accountable Care, National Healthcare Group, Singapore; 5 Woodlands Health, Singapore

**Keywords:** population health, global population health management, HealthierSG

Healthcare systems across high-income economies [1] are plagued by similar challenges, including an aging population, the increasing burden of chronic disease, and spiraling healthcare costs. David Kindig and Greg Stoddart defined population health as “the health outcomes of a group of individuals, including the distribution of such outcomes within the group” [2], and there has been a deliberate move towards population health management to address much of these challenges. Population health management efforts do not just target populations that are sick or the hospitalized (“patient” population). It also involves ensuring that healthy populations receive preventive services to avoid onset of disease. In this editorial we consider this global move towards population health management in the context of Singapore’s HealthierSG initiative. HealthierSG was announced by the Minister for Health in March 2022 and is a step forward in Singapore’s population health journey [3].

## Key Aspects of Population Health Management

Globally, there has been a shift away from treating patients episodically in acute hospitals. There is an increasing focus on looking after the population at large, keeping them as healthy as possible across their lifespan in the community. This requires considerable transformation of the healthcare delivery system, and we are seeing many systems growing services in the community and expanding transitional care services to help patients stay out of the hospital. Primary care plays a critical role, with multi-disciplinary teams of care managers, pharmacists, nurses, social workers, and allied health staff, rising to the challenge of providing preventive care.

Since 2013, in Norway, much has been done to strengthen primary care and keep older persons in the community. Municipalities provide primary care and social services, while hospitals, which are run by the state, provide acute services. To avoid delivery of fragmented care, an innovative programme called the “Holistic Continuity of Patient Care” (HCPC) was designed. Integrated clinical pathways were developed to build collaboration between hospitals and municipalities so they work together towards better outcomes, such as reduced hospitalization and greater patient empowerment. A recent study showed that while primary care costs were higher in HCPC, costs associated with hospital and nursing home care were lower. Better outcomes were shown across various domains, including “enjoyment of life’, “psychological well-being”, and “social relationships and participation” [4].

Integrated care has caught on in other parts of Europe as well. More recently, in July 2022, across England, a total of 42 Integrated Care Systems (ICS) have sprung up to address similar health and social challenges. Key features of the ICS include provider collaborative and local partnerships across sectors that aim to serve the area [5].

Provider-reimbursed methodology for care is also undergoing an overhaul as value-based care has gained momentum. Linking payment with quality of care instead of solely on volume of services delivered motivate providers to deliver care more efficiently and effectively. In the United States, National health spending is projected to reach $6.2 trillion by 2028, growing at an average annual rate of 5.4% [6]. This unsustainable growth rate combined with the need to improve the quality of healthcare saw the development of Accountable Care Organisations (ACOs). ACOs bring together a variety of providers, including community providers, that assume responsibility for both cost and quality of the population they serve [7]. Today, across the United States, there are 483 Medicare ACOs that serve 11 million beneficiaries [8]. Quality indicators measure ACO performance as a whole, and determine shared savings and other incentives for physicians, thus promoting better coordination of care. The Medicare Shared Savings Program (MSSP) which started in 2012, allows ACOs to choose various tracks which offer various levels of shared risk subject to meeting quality performance standards [9]. In 2020, it was reported that since its commencement, ACOs have saved Medicare $4.7 billion in net savings [10]. Successful ACOs concurrently employ multiple population health management strategies to achieve their aims, including the strengthening of primary care services, and targeting programmes at high-utilizers and high-cost patients [11].

Bundled payments have been introduced in many health systems worldwide as well. Bundled payment involves paying for end-to-end service within an entire episode of care instead of each individual component of test, procedure and treatment. In Taiwan, in a study looking at breast cancer care, bundled payments, compared with regular programmes could lead to better outcomes and cost control in the long run [12].

Many healthcare systems today invest in electronic medical records (EMR) which enable timely information and care coordination, including across provider settings. Health information exchanges have been developed to allow for disparate EMR systems to connect. The Statewide Health Information Network for New York (SHIN-NY) is an example of an effort that allows different large hospital systems across the state of New York to work together to support team-based decision making and to coordinate care for patients [13].

Data analytics using data that includes demographic information, clinical information, and social determinants of health, enable a better understanding of population needs, tailoring of interventions to specific at-risk groups, and improved outcomes. The Johns Hopkins Adjusted Clinical Groups (ACG) System is a risk stratification tool that has been used in many healthcare systems around the world. One study found it particularly useful in the identification of patients with very complex needs in the primary care setting that would benefit care management [14].

## Singapore’s Context: Healthier SG

Singapore is well known for delivering good healthcare outcomes at a relatively low cost, with one of the highest life expectancies in the world and a national healthcare expenditure of only 4% of GDP [15,16]. The healthcare system is anchored by three large publicly-funded integrated health systems which deliver the majority of acute care. The delivery of community and primary care is much more fragmented, with multiple stakeholders in the non-profit and private sectors playing a part. With a rapidly ageing population and rising chronic disease prevalence, very much like the rest of the developed world, Singapore has to make a transformational shift towards ***managing the health of its population*** rather than just ***managing the healthcare of patients*** in times of acute crisis. Healthier SG represents the evolution of a healthcare system that has been traditionally focused on delivering high-quality, efficient and affordable acute care via its publicly-funded hospitals, to one that brings together multiple care providers in the ecosystem to deliver holistic, longitudinal care to the Singaporean population. The key aspects of Healthier SG mirror the key aspects of population health mentioned above.

First, an important focus of Healthier SG is to change the way care is delivered from a largely episodic basis today, to a more longitudinal basis.

Only three in five Singaporeans have a regular family doctor today [3]. Healthier SG seeks to persuade Singaporeans to anchor themselves with a primary care doctor and attend regular check-ins for preventive care and chronic disease management. It mobilises primary care practitioners to take on these preventive health roles and encourages community partnerships to support “social prescriptions” (such as exercise programs) that drive lifestyle adjustments.

Second, under Healthier SG, three public Regional Health Systems (RHS) will play the role of regional health managers in integrating care longitudinally and across different providers [17]. Each RHS, which already consists of several general and specialty hospitals, ambulatory clinics and primary care clinics known as polyclinics, has also been building strong networks with private primary care practitioners and community partners. As an example, our RHS, the National Healthcare Group, is scaling up Communities of Care in the Central-North region. These are networks of partners that comprise community health teams from the acute hospital, primary care practitioners, and community partners, to deliver place-based, person-centered care in local neighbourhoods [18].

Third, RHSs will soon be funded by the Ministry of Health on a capitated basis instead of a workload basis [3]. Each RHS will be accountable for the health outcomes and costs of between 1.2 million to 1.5 million Singaporeans living in its region. Capitated funding spurs RHSs to work with its partners to implement population health programs that deliver quality outcomes at a sustainable cost.

Fourth, in recognition of the importance of data and IT integration in enabling population health, the Ministry of Health is exploring a new Health Information Bill in the next few years [3]. Today, all the RHSs and nearly 60 percent of private primary care practitioners already have access to summarised patient health records on the National Electronic Health Record system [19]. The upcoming Health Information Bill will build on this foundation to further drive the safe and secured sharing of healthcare data.

## Opportunities and challenges

The public healthcare system in Singapore has undergone a number of significant re-organisations since 2000. Each shift promised to deliver better results, but the fragmentation of financing and care has hampered success. With HealthierSG, we may have finally arrived at a tipping point to facilitate change.

With the geographical assignment of health outcomes and costs to the RHS, we have a clear opportunity to further develop place-based care. To succeed, RHSs must work to build strong networks of care and accountability frameworks with partners, based on a shared vision. A shift away from acute hospital- or even health-centricity is essential to better promote inter-sectoral partnerships [20].

While many inter-sectoral pilots have begun, many are still in their infancy. Due to differences in culture, structure and resource availability, successful collaborations across social and healthcare sectors are challenging to forge. Learning-by-doing can help us gain more knowledge on establishing successful networks and support future efforts.

Paying providers a fixed pre-determined per-person payment provides the opportunity to align incentives within our health system to reduce waste. However, it risks under delivering on services [21]. To counter this, studies have proposed blended payment models, which could include a fee-for-service payment component for prioritised health conditions or high-cost services that pose higher-risk of under-provision. Carefully designed financing arrangements linking payments to high quality care and outcomes can guide provider behavior toward population health care objectives [22].

To maximise population-wide health outcomes, decisions must be made around expanding the reach of population health interventions. However, beyond the effectiveness of individual interventions, the healthcare system’s capacity to facilitate change, is just as crucial [23]. As an example, while there are now efforts to accelerate the sharing of patient data within the healthcare sector, a complete picture of a patient’s physical and psychosocial health will require information from social care agencies. Further work is needed at the system-level to accelerate the impact of our population health interventions.

## Conclusion

As we look across the globe, there are numerous examples of innovative population health management initiatives employed to address various healthcare challenges. While there is certainly no one-size-fits-all solution, there is still ample opportunity to learn from one another so as to keep the populations we care for as healthy as possible.
